# Association of cerebrospinal fluid neurogranin levels with cognition and neurodegeneration in Alzheimer’s disease

**DOI:** 10.18632/aging.103211

**Published:** 2020-05-18

**Authors:** Mei Xue, Fu-Rong Sun, Ya-Nan Ou, Xue-Ning Shen, Hong-Qi Li, Yu-Yuan Huang, Qiang Dong, Lan Tan, Jin-Tai Yu, Alzheimer’s Disease Neuroimaging Initiative

**Affiliations:** 1 Department of Neurology, Qingdao Municipal Hospital, Qingdao University, Qingdao, China; 2 Department of Neurology and Institute of Neurology, Huashan Hospital, Shanghai Medical College, Fudan University, Shanghai, China

**Keywords:** Alzheimer’s disease, neurogranin, cerebrospinal fluid, biomarker, mild cognitive impairment

## Abstract

Accumulating data suggest cerebrospinal fluid (CSF) neurogranin (Ng) as a potential biomarker for cognitive decline and neurodegeneration in Alzheimer disease (AD). To investigate whether the CSF Ng can be used for diagnosis, prognosis, and monitoring of AD, we examined 111 cognitively normal (CN) controls, 193 mild cognitive impairment (MCI) patients and 95 AD patients in the Alzheimer’s Disease Neuroimaging Initiative (ADNI) cohort. Correlations were tested between baseline CSF Ng levels and baseline core AD biomarkers and longitudinal glucose metabolism, brain atrophy and cognitive decline. We detected that CSF Ng levels increased with disease severity, and correlated with phosphorylated tau and total tau levels within each diagnostic group. High baseline CSF Ng levels correlated with longitudinal reductions in cortical glucose metabolism within each diagnostic group and hippocampal volume within MCI group during follow-up. In addition, high baseline CSF Ng levels correlated with cognitive decline as reflected by decreased cognitive scale scores. The CSF Ng levels predicted future cognitive impairment (adjusted hazard ratio:3.66, 95%CI: 1.74-7.70, P = 0.001) in CN controls. These data demonstrate that CSF Ng offers diagnostic utility for AD and predicts future cognitive impairment in CN individuals and, therefore, may be a useful addition to the current AD biomarkers.

## INTRODUCTION

Alzheimer’s disease (AD) is a multifactorial age-related neurodegenerative disease whose pathology starts decades before the clinical symptoms appear [1]. Pathological biomarker research made it possible to diagnose the disease at the preclinical stage and predict cognitive decline before the onset of dementia [2]. In 2018, the National Institute on Aging and Alzheimer’s Association (NIA-AA) created a new “ATN” scheme for defining and staging the disease across its entire spectrum. The scheme recognizes three general groups of biomarkers: biomarkers of β-amyloid (Aβ) deposition are labeled “A”; biomarkers of pathologic tau are labeled “T”; biomarkers of neurodegeneration are labeled “N”. Besides the biomarkers mentioned above, new biomarkers can be added to the three existing ATN groups, and new biomarker groups reflecting different aspects of pathology can be added beyond ATN when they become available. Indeed, the NIA-AA research framework suggested that neurogranin (Ng) in cerebrospinal fluid (CSF) as a marker of synaptic degeneration should be investigated for potential added value in predicting cognitive decline [3].

Ng is a calmodulin-binding postsynaptic protein and plays a critical role in plasticity, synapse repair, and long-term potentiation [4, 5]. It is expressed within dendritic spines on postsynaptic neurons [6]. Increased concentrations of CSF Ng signify a loss of synaptic integrity [7, 8]. Synaptic dysfunction, an early and prominent pathologic feature of AD [9, 10], correlates with cognitive deficits and occurs prior to neuronal degeneration [11–13]. Thus, a reduction of synaptic protein such as Ng in the brain relates to synaptic dysfunction and the CSF levels of Ng can be used for disease diagnosis and prognosis. Previous studies suggest that CSF Ng levels are elevated in AD [14] and correlate with amyloid load, brain atrophy and cognitive decline [15, 16]. Increased CSF levels of Ng are specific to AD and not seen in other neurodegenerative diseases [17, 18].

In the present study, we present results on CSF Ng in the Alzheimer’s Disease Neuroimaging Initiative (ANDI) cohort of cognitively normal (CN) controls, patients with mild cognitive impairment (MCI) and patients with AD. We tested the specific hypotheses that the CSF Ng levels were altered in patients with AD and had diagnostic utility for AD, that the CSF Ng correlated with AD core biomarkers (CSF Aβ, phosphorylated tau (p-tau) and total tau (t-tau)), cognitive decline and imaging evidence of neurodegeneration and that the CSF Ng levels predicted future cognitive impairment at the early asymptomatic stage.

## RESULTS

### Basic characteristics

The baseline characteristics of participants are shown in Table 1. A total of 399 individuals were included in this study (111 with CN diagnosis, 193 with MCI diagnosis and 95 with AD diagnosis). There were no significant differences in age and educational level across the three groups. The MCI group had fewer females than the CN group (P = 0.004). Significant differences in the frequency of the APOE ε4 allele were detected across the three groups (AD > MCI > CN, P<0.001). As expected, there were significant differences in CSF Aβ, p-tau and t-tau levels and cognitive scale scores across the three groups (P <0.001). The subjects with AD had the lowest CSF Aβ levels, the highest CSF t-tau and p-tau levels, and the lowest ADNI_MEM and ADNI_EF scores. The CSF Ng levels did not differ by age in non-demented participants (P = 0.400). In the Aβ+ group, mean CSF Ng levels were higher in female individuals compared with those in male individuals (P=0.003) (Supplementary Figure 1). Clinical follow-up data were available for 109 subjects with CN (76 remained stable, 33 progressed to MCI) and 187 subjects with MCI (80 remained stable (stable MCI, sMCI), 107 progressed to AD (progressive MCI, pMCI)). Details of the information for each group are reported in the Supplementary Table 1 and Table 2.

**Table 1:** Baseline characteristics of the study participants.

**Characteristics**	**CN (n=111)**	**MCI (n=193)**	**AD (n=95)**
Age ^a^, mean (SD) years	75.6 (5.2)	74.4 (7.5)	74.5 (7.9)
Female ^b,e^, N (%)	55 (49.5)	63 (32.6)	42 (44.2)
Education ^a^, mean (SD) years	15.8 (2.8)	15.7 (3.0)	14.5 (3.2)
APOE ε4 carriers ^b,c,d,e^, N (%)	27 (24.3)	103 (53.4)	67 (70.5)
ADNI_MEM ^a,c,d,e^, mean (SD)	0.94 (0.50)	-0.14 (0.57)	-0.85 (0.53)
ADNI_EF ^a,c,d,e^, mean (SD)	0.64 (0.60)	-0.05 (0.75)	-0.99 (0.89)
CSF Ng ^a,d,e^, mean (SD), pg/mL	351.5 (292.2)	491.7 (350.8)	551.3 (325.8)
CSF Aβ ^a,c,d,e^, mean (SD), pg/mL	207.2 (53.0)	165.1 (51.7)	143.0 (37.0)
CSF p-tau ^a,c,d,e^, mean, (SD), pg/mL	25.5 (14.8)	35.8 (18.5)	41.5 (19.6)
CSF t-tau ^a,c,d,e^, mean, (SD), pg/mL	68.9 (29.2)	102.3 (59.6)	121.6 (55.9)

Abbreviations: CN, cognitively normal; MCI, mild cognitive impairment; AD, Alzheimer’s disease; APOE, apolipoprotein E; ADNI, Alzheimer’s Disease Neuroimaging Initiative; ADNI_MEM, memory domain summary score; ADNI_EF, executive domain summary score; CSF: cerebrospinal fluid; Ng, neurogranin; Aβ, amyloid-β; t-tau, total tau; p-tau, phosphorylated tau.

^a^Kruskal-Wallis test.

^b^Chi-square (χ2) tests.

^c^Significant differences between AD and MCI (p < 0.05).

^d^Significant differences between AD and CN (p < 0.05).

^e^Significant differences between MCI and CN (p < 0.05).

### Diagnostic utility of CSF Ng in AD

**Figure 1:**
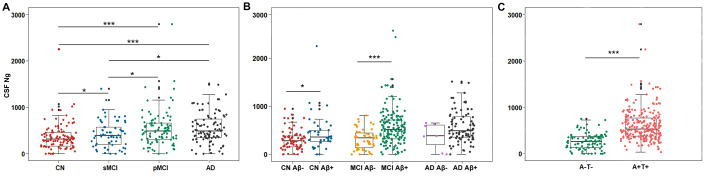
**Scatterplots of CSF Ng levels by clinical diagnosis and biological status.** (**A**) Mean CSF Ng levels were higher in AD subjects compared with sMCI subjects (P = 0.011) or CN controls (P < 0.001). Mean CSF Ng levels were higher in pMCI subjects compared with sMCI subjects (P = 0.028) or CN controls (P < 0.001). Mean CSF Ng levels were higher in sMCI subjects compared with CN controls (P = 0.042). (**B**) When comparing by Aβ status, Ng values were differentially increased in Aβ+ CN (P = 0.032) and Aβ+ MCI individuals (P < 0.001), whereas in the dementia stage, Ng levels were elevated regardless of Aβ status (P = 0.243). (**C**) Mean CSF Ng levels were higher in those with A+T+ (Mean [SD]: 608.7 [345.0] pg/mL, n = 230) compared with those with A-T- (Mean [SD]: 260.5 [175.6] pg/mL; n = 99) (P < 0.001). Mann-Whitney U test/Kruskal-Wallis test was used for all group comparisons. * p<0.05, *** p<0.001. Abbreviations: CN, cognitively normal; MCI, mild cognitive impairment; sMCI, stable MCI; pMCI, progressive MCI, MCI progressing to dementia due to AD; AD, Alzheimer’s disease; CSF: cerebrospinal fluid; Ng, neurogranin; Aβ, amyloid-β; A-, amyloid-β negative (CSF Aβ>192 pg/mL); A+, amyloid-β positive (CSF Aβ<192 pg/mL); T-, tau negative (CSF p-tau<23 pg/mL); T+, tau positive (CSF p-tau>23 pg/mL).

**Figure 2:**
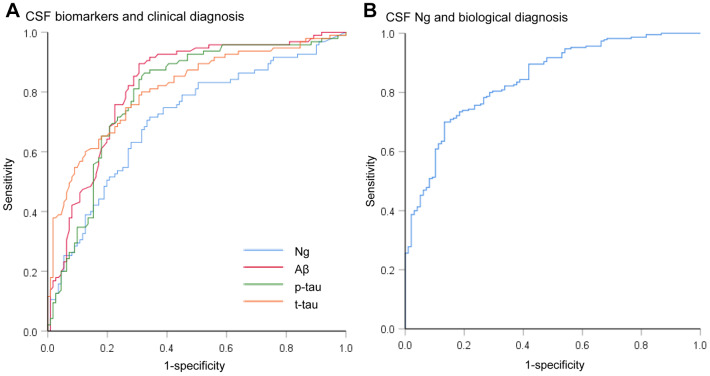
**Receiver operating characteristic curves for the diagnostic utility of CSF biomarkers.** (**A**) Receiver operating characteristic curves for the diagnostic utility of CSF biomarkers in differentiating AD from controls by clinical diagnosis (AD versus CN). The diagnostic accuracy (area under the receiver operating characteristic curve [AUC]) of CSF Ng in differentiating patients with AD from controls was comparable to that of the core CSF biomarkers. (**B**) Receiver operating characteristic curves for the diagnostic utility of CSF Ng in differentiating AD from controls by biological status (A+T+ versus A-T-). Abbreviations: CSF: cerebrospinal fluid; Ng, neurogranin; Aβ, amyloid-β; p-tau, phosphorylated tau; t-tau, total tau.

Mean CSF Ng levels were higher in AD subjects compared with sMCI subjects (P = 0.011) or CN controls (P < 0.001). Mean CSF Ng levels were higher in pMCI subjects compared with sMCI subjects (P = 0.028) or CN controls (P < 0.001). Mean CSF Ng levels were higher in sMCI subjects compared with CN controls (P = 0.042) (Figure 1A). When comparing by Aβ status, Ng values were differentially increased in Aβ+ CN (P = 0.032) and Aβ+ MCI individuals (P < 0.001), whereas in the dementia stage, Ng levels were elevated regardless of Aβ status (P = 0.243) (Figure 1B). Similarly, mean CSF Ng levels were higher in those with A+T+ (Mean [SD]: 608.7 [345.0] pg/mL, n = 230) compared with those with A-T- (Mean [SD]: 260.5 [175.6] pg/mL; n = 99) (P < 0.001) (Figure 1C). The diagnostic accuracy (area under the receiver operating characteristic curve [AUC]) of CSF Ng in differentiating patients with AD from CN was comparable to that of the core CSF biomarkers (Figure 2A). The mean (SD) AUC was 0.82 (0.03) for Aβ, 0.79 (0.03) for p-tau, 0.81 (0.03) for t-tau, and 0.71 (0.04) for Ng. The CSF Ng levels also had diagnostic accuracy in differentiating patients with A+T+ from A-T-, the mean (SD) AUC was 0.85 (0.02) (Figure 2B).

### Correlations of CSF Ng with core CSF biomarkers, imaging markers and cognitive scores

The CSF Ng levels correlated with CSF p-tau (β = 0.61, β = 0.58 and β = 0.72) and t-tau (β = 0.50, β = 0.74 and β = 0.81) levels in subjects with CN, MCI and AD, respectively (P < 0.001). No correlations were observed between CSF Ng levels and CSF Aβ levels in patients with AD (β = -0.13, P = 0.117) or CN controls (β = -0.12, P = 0.163). The CSF Ng levels negatively correlated with CSF Aβ levels in subjects with MCI (β = -0.22, P = 0.001). Significant association of baseline CSF Ng levels and ^18^F-Fluorodeoxyglucose positron emission tomography (FDG-PET) was identified in subjects with CN (β = -0.06, P = 0.002), MCI (β = -0.04, P = 0.017) and AD (β = -0.15, P = 0.009). The CSF Ng levels were negatively correlated with hippocampal volume in subjects with MCI (β = -0.02, P = 0.017). The CSF Ng levels also related to the decline of ADNI_MEM (β = -0.05, P<0.001 and β = -0.07, P = 0.044) and ADNI_EF (β = -0.04, P = 0.013 and β = -0.10, P = 0.008) scores in subjects with MCI and AD (Table 2).

**Table 2:** Correlations of CSF Ng with core CSF biomarkers, imaging markers and cognitive scores.^a^

**Model**	**CN**		**MCI**		**AD**	
**Cross-sectional^b^**	**β (95%CI)**	**P**	**β (95%CI)**	**P**	**β (95%CI)**	**P**
CSF Aβ	-0.13 (-0.26, -0.04)	0.117	-0.22 (-0.28, -0.16)	0.001	-0.12 (-0.20, -0.04)	0.163
CSF p-tau	0.61 (0.54, 0.68)	<0.001	0.58 (0.52, 0.64)	<0.001	0.72 (0.65, 0.79)	<0.001
CSF t-tau	0.50 (0.42, 0.58)	<0.001	0.74 (0.69, 0.79)	<0.001	0.81 (0.74, 0.88)	<0.001
**Longitudinal^c^**	**β (95%CI)**	**P**	**β (95%CI)**	**P**	**β (95%CI)**	**P**
FDG-PET^d^	-0.06 (-0.08, -0.04)	0.002	-0.04 (-0.06, -0.02)	0.017	-0.15 (-0.20, -0.10)	0.009
Hippocampal volume^e^	-0.01 (-0.02, -0.00)	0.091	-0.02 (-0.03, -0.01)	0.017	-0.01 (-0.02, -0.00)	0.470
ADNI_MEM	-0.03 (-0.04, -0.02)	0.063	-0.05 (-0.07, -0.03)	<0.001	-0.07 (-0.10, -0.04)	0.044
ADNI_EF	-0.02 (-0.03, -0.01)	0.254	-0.04 (-0.06, -0.02)	0.013	-0.10 (-0.13, -0.07)	0.008

Abbreviations: CSF, cerebrospinal fluid; Ng, neurogranin; Aβ, amyloid-β; p-tau, phosphorylated tau; t-tau, total tau; FDG-PET, ^18^F-Fluorodeoxyglucose positron emission tomography; ADNI, Alzheimer’s Disease Neuroimaging Initiative; ADNI_MEM, memory domain summary score; ADNI_EF, executive domain summary score.

^a^All models are adjusted for age, sex, educational level, APOE ε4 genotype and intracranial volume (for Hippocampus only). All variables were z-scale transformed to normalize the distributions.

^b^Multiple regression model.

^c^Mixed effects linear model.

^d^Individuals who underwent positron emission tomography (n = 193) included CN controls (n = 53), subjects with MCI (n = 95) and patients with AD (n = 45).

^e^Individuals who underwent magnetic resonance imaging (n = 338) included CN controls (n = 105), subjects with MCI (n = 162) and patients with AD (n = 71).

### Ability of CSF Ng levels to predict future cognitive impairment

**Figure 3:**
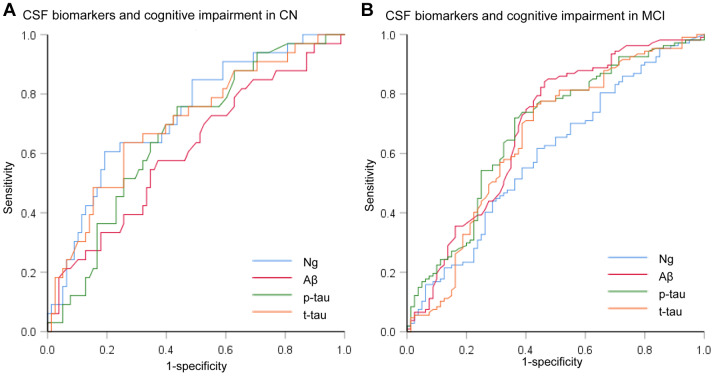
**Receiver operating characteristic curves for the predictive utility of CSF biomarkers.** (**A**) Receiver operating characteristic curves for predicting future cognitive impairment in cognitively normal controls over time (CN to MCI). (**B**) Receiver operating characteristic curves for predicting future cognitive impairment in MCI subjects over time (MCI to AD). Abbreviations: CSF: cerebrospinal fluid; Ng, neurogranin; Aβ, amyloid-β; p-tau, phosphorylated tau; t-tau, total tau.

**Figure 4:**
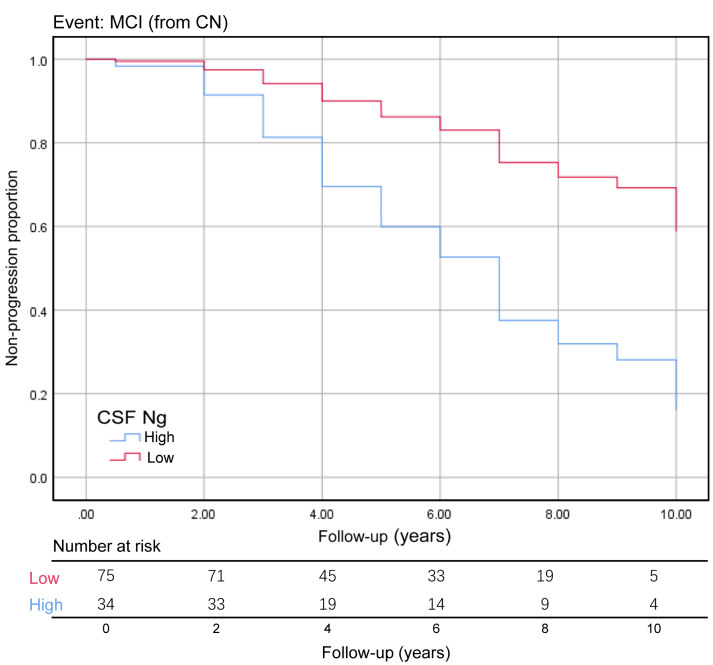
**Baseline CSF Ng levels as the predictor of conversion from CN to MCI.** The Kaplan-Meier curve showed the predictive value of the CSF Ng for progression from cognitively normal to MCI. The CSF Ng was analyzed as categorical variables (dichotomized at 389 pg/mL), and analysis was adjusted for age, sex, educational level, and APOE ε4 genotype. Abbreviations: CN, cognitively normal; MCI, mild cognitive impairment; CSF: cerebrospinal fluid; Ng, neurogranin.

We assessed the ability of CSF biomarkers to predict future cognitive impairment in cognitively normal controls over time. The CSF Ng levels provided higher predictive accuracy than the core CSF biomarkers (Figure 3A). The mean (SD) AUC was 0.73 (0.05) for Ng, 0.62 (0.06) for Aβ, 0.67 (0.05) for p-tau, and 0.71 (0.06) for t-tau. The cox proportional hazards regression models were also developed to estimate the predictive value of CSF biomarkers (as categorical variables) in the conversion risk from CN to MCI (Table 3). After adjustment for age, sex, educational level, and APOE ε4 genotype, only CSF Ng showed the ability to predict cognitive impairment. Compared with the low level of CSF Ng, high level was associated with 3.66-fold increased risk of MCI (95% CI: 1.74-7.70, P = 0.001) (Figure 4). This association was almost identical after additional adjustment for CSF Aβ (Hazard ratio: 3.31, 95%CI: 1.52-7.25, P = 0.003). Furthermore, we examined whether CSF biomarkers predicted conversion from MCI to AD over time. The mean (SD) AUC was 0.69 (0.04) for Aβ, 0.68 (0.04) for p-tau, 0.65 (0.04) for t-tau, and 0.60 (0.04) for Ng in differentiating patients with pMCI from sMCI (Figure 3B). The cox proportional risk regression model showed, with the exception of CSF Ng, all CSF biomarkers predicted conversion from MCI to AD during follow-up (Supplementary Table 3).

**Table 3:** CSF biomarker variables as predictors of time to conversion from CN to MCI.^a^

**Biomarker**	**Unadjusted hazard ratio (95% CI)**	**P**	**Adjusted hazard ratio (95% CI)^b^**	**P**
CSF Ng	3.34 (1.66, 6.73)	0.001	3.66 (1.74, 7.70)	0.001
CSF Aβ	0.42 (0.21, 0.84)	0.014	0.52 (0.24, 1.10)	0.085
CSF p-tau	0.73 (0.52, 1.03)	0.076	0.77 (0.53, 1.11)	0.159
CSF t-tau	0.70 (0.49, 1.01)	0.057	0.70 (0.46, 1.04)	0.079

Abbreviations: CN, cognitively normal; MCI, mild cognitive impairment; CSF: cerebrospinal fluid; Ng, neurogranin; Aβ, amyloid-β; t-tau, total tau; p-tau, phosphorylated tau.

^a^Cox proportional hazards regression models tested the effects of CSF biomarkers on the conversion rate from CN to MCI. The CSF biomarker measures were analyzed as categorical variables (dichotomized at the cut-off value; CSF Ng: 389 pg/mL, CSF Aβ: 192 pg/mL, CSF p-tau: 23 pg/mL, CSF t-tau: 93 pg/mL).

^b^Models are adjusted for age, sex, educational level, APOE ε4 genotype.

## DISCUSSION

In this study, we found that (1) CSF Ng levels were elevated in AD, pMCI and sMCI groups compared with CN group and the diagnostic utility of CSF Ng levels differentiating patients with AD from CN controls was comparable to that of core CSF biomarkers; (2) CSF Ng levels associated with CSF p-tau and t-tau levels within each diagnostic group and with CSF Aβ levels within MCI group; (3) high baseline CSF Ng levels correlated with longitudinal reduction of cognitive scale scores within MCI and AD groups, decreased cortical glucose metabolism within each diagnostic group, and hippocampal volume atrophy within MCI group; (4) CSF Ng levels predicted conversion from CN to MCI. Taken together, these findings suggest that CSF Ng is a very early and potentially presymptomatic biomarker for AD. This biomarker may be helpful in AD diagnosis, predicting disease progression and staging severity of AD even in its preclinical stage. Our study also provided clues to how Ng participated in the pathophysiological process in AD, to monitor drug effects on synaptic degeneration in clinical treatment trials, and provided evidence for drug development.

We found that mean CSF Ng levels were higher in female individuals compared with those in male individuals in the Aβ+ group. A potential explanation of this difference maybe that sex is a crucial variable in disease heterogeneity of AD. The cumulative evidence indicates that women exhibit steeper cognitive decline and higher rates of brain atrophy after diagnosis of MCI or AD [19]. Women may show more serious synaptic degeneration after the pathology of AD (accumulation of Aβ plaques) appear. CSF levels of Ng are significantly increased in subjects with AD as compared to subjects with sMCI and CN controls. This is consistent with previous reports in both ADNI study [14, 20, 21] and other cohorts [15, 22–24]. There was no significant difference between AD and pMCI groups, whereas, CSF Ng levels are higher in patients with MCI who progress to AD than in patients with MCI who remain stable. A recent study showed that elevated Ng levels were associated with cognitive decline in participants with MCI [25]. Thus, the CSF Ng levels appear to have a diagnostic as well as a prognostic value. Further, CSF Ng levels were differentially increased in Aβ+ individuals compared with that in Aβ- individuals within CN and MCI groups. Amyloid plaques are toxic to the brain parenchyma, inducing various processes responsible for synaptic loss [26]. Therefore, Aβ+ did have an effect on Ng levels. The mechanism of Ng secretion from neurons to CSF is currently unknown, but enzymatic cleavage of Ng may be of relevance [27]. C-terminal Ng is increased in CSF in AD, which strengthens the potential of neurogranin as an AD CSF biomarker [24].

We observed positive correlations between CSF Ng levels and CSF p-tau and t-tau levels in each diagnostic group and negative correlations between CSF Ng levels and CSF Aβ levels in MCI group. This relationship gives support to the utility of CSF Ng as a biomarker, for it is sensitive to AD-related biological changes. There is evidence that tau pathology is involved in synapse degeneration and contributes to cognitive decline [28, 29]. The absence of correlation between CSF Ng levels and CSF Aβ levels showed that there were no correlations between both the synapse loss and clinical stage and the amount of amyloid plaques [30–32]. AD is a continuum pathology, there is no clear discrimination in amyloid plaque numbers between patients with AD and cognitively intact elderly who died from other reasons [33]. The current data also allowed us to examine the associations of CSF Ng levels with two other key biomarkers for AD: hippocampal atrophy measured by volumetric MRI and cortical glucose metabolism assessed by FDG-PET. We found a relationship between high CSF Ng levels and reduced cortical glucose metabolism. Associations between high CSF Ng levels and increased rate of hippocampal atrophy only present in MCI group.

Logistic regression analysis was used to assess the impact of CSF analytes on risk for disease progression. The AUC (reflect predictive probabilities of the logistic regression models) of the CSF Ng model was great in predicting progression from CN to MCI. High CSF Ng levels are strongly associated with risk of MCI in cognitive normal participants, suggesting that CSF Ng is a marker of clinical progression in asymptomatic population. The CSF Ng levels reflect synaptic loss in a very early stage but not in later stages of the AD spectrum. Our data showed that synaptic dysfunction was, to some extent, involved in AD pathophysiology. Low CSF Aβ is considered a biomarker of an individual in the Alzheimer’s continuum [3]. Thus, we examined whether CSF Aβ was an effect modifier in the analysis of CSF Ng for risk of MCI. The result indicated that the association between elevated CSF Ng levels and risk of MCI was independent of CSF Aβ, suggesting that elevated CSF Ng levels might be a risk factor for cognitive decline for those on the AD pathway and for those who are not. A previous study suggests that elevated CSF neurofilament light (NFL) levels but not CSF Ng levels are a risk factor for MCI [34]. This finding contradicts our results and a potential explanation may be a difference of the participants between the two studies. Their study included the community-based population, but our study used the ADNI cohort.

There were several limitations of our study. Firstly, CSF biomarkers included in the cox regression models as dichotomous variables might conceal an underlying continuum. Secondly, the newly published NIA-AA criteria conducts an ATN classification system and the ATN system is flexible in that new biomarkers can be added to the three existing ATN groups [3]. Whether Ng can be the preferred “N” in the ATN groups need to be further explored. Finally, the restricted sample selection in the ADNI should be taken into consideration for interpreting the data.

Taken together, our findings suggest that the CSF Ng can be used as a biomarker for synaptic pathology in AD, and CSF Ng is a valuable biomarker of early neurodegeneration. In addition to the “core CSF biomarkers” Aβ, p-tau, and t-tau, the CSF Ng might have added value.

## MATERIALS AND METHODS

### Participants

Data used in this article were obtained from the ADNI database (adni.loni.usc.edu) [35]. ADNI was launched in 2003 as a public–private partnership, and the principal investigator of this initiative is Michael W. Weiner, MD, VA Medical Center and University of California, San Francisco. The primary goal of ADNI has been to test whether serial magnetic resonance imaging, PET and additional biological markers and clinical and neuropsychological assessments can be combined to measure the progression of MCI and early clinical AD. To date, it has three phases: ADNI1, ADNI GO and ADNI2, consisting of individuals recruited from over 50 sites across the USA and Canada. All individuals included were between the ages of 55 and 90 years, had completed at least 6 years of education, were considered to be CN, MCI or AD dementia diagnosed individuals, and underwent serial evaluations of functional, biomedical, neuropsychological and clinical status at various intervals [36]. For up to date information, see http://www.adni-info.org.

We included all CN controls, MCI patients and AD patients with available baseline CSF Ng samples. CN controls had Mini-Mental State Examination (MMSE) score between 25 and 30; clinical dementia rating (CDR) score of 0. CN controls were grouped into those that remained cognitively stable for at least 1 follow-up and those who progressed to MCI during follow-up. Subjects with MCI had MMSE score between 24 and 30; CDR score of 0.5; objective memory loss as shown on scores on delayed recall of the Wechsler memory scale logical memory II [> 1 standard deviations (SD) below the normal mean]; preserved activities of daily living, and the absence of dementia. In our study, patients with MCI were grouped into those that remained cognitively stable for at least 1 follow-up (sMCI) and those who progressed to AD dementia during follow-up (pMCI). Patients with AD fulfilled the National Institute of Neurological Communicative Disorders and Stroke-Alzheimer Disease and Related Disorders Association criteria for probable AD, had MMSE scores between 20-26 and a Clinical Dementia Rating scale of 0.5 or 1.0. As to “ATN” categories: amyloid positive and negative (A+ versus A-) were separated by a cutoff value of 192 pg/mL for CSF Aβ level; tau pathology positive and negative (T+ versus T-) were separated by a cutoff value of 23 pg/mL for CSF p-tau level [37–40].

### CSF measurements

CSF Ng was analyzed by electrochemiluminescence technology using Ng7, which is a monoclonal antibody specific for Ng, as coating antibody and polyclonal Ng anti-rabbit (ab 23570, Upstate) as detector antibody [16]. Values are given as pg/mL. CSF Aβ, p-tau and t-tau were measured using the multiplex xMAP Luminex platform (Luminex Corp, Austin, TX) with Innogenetics (INNO-BIA AlzBio3; Ghent, Belgium; for research use–only reagents) immunoassay kit-based reagents. Values are given in pg/mL for both tau and Aβ [40].

### Neuroimaging and cognition

Magnetic resonance (MR) images were collected from a variety of 1.5/3.0 Tesla MR system, using protocols optimized for each MR scanner. The FreeSurfer pipeline was used to generate hippocampus estimates [41]. We used averaged volume measurements for the right and left hippocampi.

Mean FDG uptake was obtained per subject within a set of predefined and previously validated regions of interest (right and left inferior temporal and lateral parietal regions, and a bilateral posterior cingulate cortex region) based on a literature as described elsewhere in detail [42]. Each subject’s summary FDG index was the mean of the region of interest relative to the mean of a pons and cerebellar vermis reference region.

Summary cognitive scores were chosen over individual cognitive tests to use more comprehensive and robust measures of domain-specific cognitive performance. Summary metric for the memory cognitive domain was ADNI-MEM (derived from: Rey Auditory Verbal Learning Test (RAVLT, 2 versions), AD Assessment Schedule-Cognition (ADAS-Cog, 3 versions), Mini-Mental State Examination (MMSE), and Logical Memory data) [43] and for the executive cognitive domain was ADNI-EF (derived from: Wechsler Adult Intelligence Scale-Revised Digit Symbol Substitution, Digit Span backwards, Trail Making Test parts A and B, animal and vegetable Category Fluency, and Clock Drawing Test) [44].

### Statistical analysis

Tests of inter-group differences were performed using Chi-square analysis for frequencies or Mann-Whitney U test/Kruskal-Wallis test for continuous measures. Linear regression models were constructed to examine the cross-sectional associations between CSF Ng levels and core CSF biomarkers (CSF Aβ, p-tau and t-tau) at baseline. Longitudinal associations between CSF Ng levels and cognitive, metabolic and structural data were assessed using linear mixed-effects model. Each CSF variable, hippocampal volume, FDG-PET and cognitive scale scores were z-scale transformed to ensure normality. Model was adjusted for age, sex, educational and APOE genotype (and adjusted for intracranial volume for hippocampal volume). Logistic regression analysis was used to assess the impact of different CSF analytes on the risk of disease progression. The receiver-operator curves and the area under the curves were derived from the predictive probabilities of the logistic regression models. Cox proportional hazard regression models access whether the CSF biomarkers (as categorical) predict cognitive impairment. The cut-off value of CSF Ng was obtained from receiver operating characteristic curve. Participants were followed up until a diagnosis of MCI/AD, death, or last follow-up visit. Time to event was defined as time from baseline CN to first visit defined as MCI/ baseline MCI to first visit defined as AD.

All tests were two-sided, statistical significance was set at P < 0.05. All statistics were performed using R 3.6.2 and IBM SPSS Statistics 25.

## Supplementary Material

Supplementary Figure 1

Supplementary Tables
