# Supplementary material for: Association of cerebrospinal fluid neurogranin levels with cognition and neurodegeneration in Alzheimer’s disease
Source: Aging (Albany NY). 2020 May 18;12(10):9365–79. doi: 10.18632/aging.103211 (PMC7288926; doi:10.18632/aging.103211)
Supplement: Supplementary Figure 1 [file aging-12-103211-s001..pdf]

SUPPLEMENTARY FIGURE

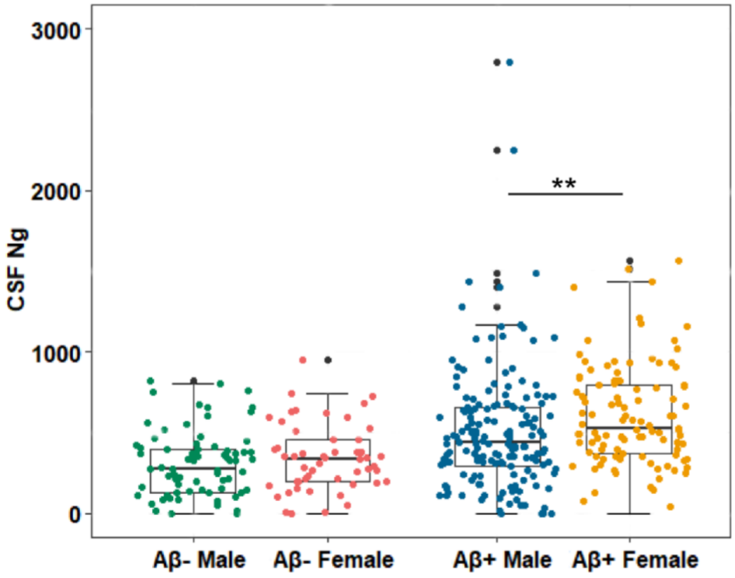

Supplementary Figure 1. Comparisons of CSF Ng levels by sex status within Aβ group. In the Aβ+ group, mean CSF Ng levels were higher in female individuals (Mean [SD]: 598.6 [318.8] pg/mL; n=108) compared with those in male individuals (Mean [SD]: 502.9 [380.6] pg/mL; n=165) (P=0.003). **P<0.01. Abbreviations: Aβ, amyloid-β; CSF, cerebrospinal fluid; Ng, neurogranin.
