# Supplementary material for: Association of cerebrospinal fluid neurogranin levels with cognition and neurodegeneration in Alzheimer’s disease
Source: Aging (Albany NY). 2020 May 18;12(10):9365–79. doi: 10.18632/aging.103211 (PMC7288926; doi:10.18632/aging.103211)
Supplement: Supplementary Tables [file aging-12-103211-s002..pdf]

SUPPLEMENTARY TABLES

Supplementary Table 1. Baseline characteristics of cognitively normal controls with longitudinal assessments.

Characteristics	Non-converters (n=76)	Converters (n=33)	P
Age, years, mean (SD)	75.6 (5.5)	75.8 (4.5)	0.666[a]
Female, N (%)	41 (53.2)	14 (41.2)	0.241[b]
Education, mean (SD) years	15.8 (2.9)	15.8 (2.7)	0.997[a]
APOE ε4, carriers, N (%)	16 (20.8)	12 (35.3)	0.105[b]
ADNI_MEM, mean (SD)	1.02 (0.49)	0.77 (0.49)	0.033[a]
ADNI_EF, mean (SD)	0.59 (0.71)	0.56 (0.71)	0.937[a]
CSF Ng, mean (SD), pg/mL	280.2 (201.0)	508.2 (379.4)	< 0.001[a]
CSF Aβ, mean (SD), pg/mL	214.6 (50.6)	192.9 (55.4)	0.065[a]
CSF p-tau, mean, (SD), pg/mL	23.6 (13.5)	29.0 (14.9)	0.007[a]
CSF t-tau, mean, (SD), pg/mL	61.7 (22.0)	82.8 (30.1)	0.001[a]

Abbreviations: APOE, apolipoprotein E; ADNI, Alzheimer's Disease Neuroimaging Initiative; ADNI_MEM, memory domain summary score; ADNI_EF, executive domain summary score; CSF: cerebrospinal fluid; Ng, neurogranin; Aβ, amyloid-β; t-tau, total tau; p-tau, phosphorylated tau.
[a]Mann-Whitney U test.
[b]Chi-square (χ2) tests.

Supplementary Table 2. Baseline characteristics of stable versus progressive MCI patients.

Characteristics	sMCI (n=80)	pMCI (n=107)	P
Age, years, mean (SD)	74.6 (7.4)	74.2 (7.5)	0.666[a]
Female, N (%)	25 (31.3)	38 (35.5)	0.542[b]
Education, mean (SD) years	15.5 (3.0)	15.9 (3.0)	0.383[a]
APOE ε4 carriers, N (%)	38 (47.5)	65 (60.7)	0.072[b]
ADNI_MEM, mean (SD)	0.05 (0.59)	-0.29 (0.50)	< 0.001[a]
ADNI_EF, mean (SD)	0.01 (0.80)	-0.14 (0.82)	0.258[a]
CSF Ng, mean (SD), pg/mL	427.4 (302.6)	539.2 (374.9)	0.028[a]
CSF Aβ, mean (SD), pg/mL	186.5 (57.1)	149.3 (41.3)	< 0.001[a]
CSF p-tau, mean, (SD), pg/mL	29.6 (15.1)	39.5 (17.6)	< 0.001[a]
CSF t-tau, mean, (SD), pg/mL	89.4 (53.4)	108.8 (51.0)	< 0.001[a]

Abbreviations: MCI, mild cognitive impairment; sMCI, stable MCI; pMCI, progressive MCI, MCI progressing to dementia due to AD; APOE, apolipoprotein E; ADNI, Alzheimer's Disease Neuroimaging Initiative; ADNI_MEM, memory domain summary score; ADNI_EF, executive domain summary score; CSF: cerebrospinal fluid; Ng, neurogranin; Aβ, amyloid-β; t-tau, total tau; p-tau, phosphorylated tau.
[a]Mann-Whitney U test.
[b]Chi-square (χ2) tests.

Supplementary Table 3. CSF biomarker variables as predictors of time to conversion from MCI to AD.[a]

Biomarker	Unadjusted hazard ratio (95% CI)	P	Adjusted hazard ratio (95% CI)[b]	P
CSF Ng	1.40 (0.95, 2.07)	0.090	0.90 (0.72, 1.11)	0.309
CSF Aβ	0.29 (0.16, 0.51)	< 0.001	0.55 (0.41, 0.75)	< 0.001
CSF p-tau	2.49 (1.54, 4.04)	< 0.001	2.31 (1.34, 3.93)	0.002
CSF t-tau	1.77 (1.20, 2.60)	0.004	1.63 (1.09, 2.44)	0.016

Abbreviations: MCI, mild cognitive impairment; AD, Alzheimer's disease; CSF: cerebrospinal fluid; Ng, neurogranin; Aβ, amyloid-β; t-tau, total tau; p-tau, phosphorylated tau.

[a]Cox proportional hazards regression models tested the effects of CSF biomarkers on the conversion rate from MCI to AD. The CSF biomarker measures were analyzed as categorical variables (dichotomized at the cut-off value: CSF Ng: 412 pg/mL, CSF Aβ: 192 pg/mL, CSF p-tau: 23 pg/mL, CSF t-tau: 93 pg/mL).

[b]Models are adjusted for age, sex, educational level, APOE ε4 genotype.
